# White’s “Universal Porte Polisher”

**Published:** 1866-01

**Authors:** 


### WHITE'S "UNIVERSAL PORTE POLISHER."

This instrument, with corundum points of various sizes and shapes, we have been using for some time as a valuable dressing instrument for gold plugs; it is a very valuable addition to our list of finishing instruments, and one that we prize very highly, and that we now would not do without, indeed no good operator can afford to be without some such instrument.

It has always been a matter of wonder to us how any one not in the active practice of the profession, can originate, and make so many useful appliances, as issue from S. S. White's dental establishment.

The remark is often made, "White has been successful;" yes he has, but it has only been attained as the result of merit, and indefatigable labor.

